# Bacterial small RNAs in the Genus *Rickettsia*

**DOI:** 10.1186/s12864-015-2293-7

**Published:** 2015-12-18

**Authors:** Casey L. C. Schroeder, Hema P. Narra, Mark Rojas, Abha Sahni, Jignesh Patel, Kamil Khanipov, Thomas G. Wood, Yuriy Fofanov, Sanjeev K. Sahni

**Affiliations:** Department of Pathology, the University of Texas Medical Branch, 301 University Blvd, Galveston, TX 77555 USA; Department of Pharmacology, the University of Texas Medical Branch, 301 University Blvd, Galveston, TX 77555 USA; Department of Biochemistry and Molecular Biology, the University of Texas Medical Branch, 301 University Blvd, Galveston, TX 77555 USA

**Keywords:** Endothelial cells, *Rickettsia*, Small RNAs, Spotted Fever, Typhus, Deep-Sequencing, Bioinformatics, SIPHT/sRNAPredict3

## Abstract

**Background:**

*Rickettsia* species are obligate intracellular Gram-negative pathogenic bacteria and the etiologic agents of diseases such as Rocky Mountain spotted fever (RMSF), Mediterranean spotted fever, epidemic typhus, and murine typhus. Genome sequencing revealed that *R. prowazekii* has ~25 % non-coding DNA, the majority of which is thought to be either “junk DNA” or pseudogenes resulting from genomic reduction. These characteristics also define other *Rickettsia* genomes. Bacterial small RNAs, whose biogenesis is predominantly attributed to either the intergenic regions (trans-acting) or to the antisense strand of an open reading frame (cis-acting), are now appreciated to be among the most important post-transcriptional regulators of bacterial virulence and growth. We hypothesize that intergenic regions in rickettsial species encode for small, non-coding RNAs (sRNAs) involved in the regulation of its transcriptome, leading to altered virulence and adaptation depending on the host niche.

**Results:**

We employed a combination of bioinformatics and in vitro approaches to explore the presence of sRNAs in a number of species within Genus *Rickettsia*. Using the sRNA Identification Protocol using High-throughput Technology (SIPHT) web interface, we predicted over 1,700 small RNAs present in the intergenic regions of 16 different strains representing 13 rickettsial species. We further characterized novel sRNAs from typhus (*R. prowazekii* and *R. typhi*) and spotted fever (*R. rickettsii* and *R. conorii*) groups for their promoters and Rho-independent terminators using Bacterial Promoter Prediction Program (BPROM) and TransTermHP prediction algorithms, respectively. Strong σ70 promoters were predicted upstream of all novel small RNAs, indicating the potential for transcriptional activity. Next, we infected human microvascular endothelial cells (HMECs) with *R. prowazekii* for 3 h and 24 h and performed Next Generation Sequencing to experimentally validate the expression of 26 sRNA candidates predicted in *R. prowazekii*. Reverse transcriptase PCR was also used to further verify the expression of six putative novel sRNA candidates in *R. prowazekii*.

**Conclusions:**

Our results yield clear evidence for the expression of novel *R. prowazekii* sRNA candidates during infection of HMECs. This is the first description of novel small RNAs for a highly pathogenic species of *Rickettsia*, which should lead to new insights into rickettsial virulence and adaptation mechanisms.

**Electronic supplementary material:**

The online version of this article (doi:10.1186/s12864-015-2293-7) contains supplementary material, which is available to authorized users.

## Background

Small noncoding RNAs (sRNAs) were first identified and described in the 1960s, but remained largely ignored until recently when they were recognized as important post-transcriptional regulators in both eukaryotic and prokaryotic organisms [1]. These sRNAs have been found in a number of pathogenic bacteria belonging to family Enterobacteriaceae, *Pseudomonas aeruginosa, Listeria monocytogenes, Streptococcus pyogenes, Clostridium perfringens,* and *Staphylococcus aureus* [2]. For example, *Helicobacter pylori,* the causative agent of chronic active, chronic persistent, and atrophic gastritis in adults and children, and implicated in a majority of duodenal and gastric ulcers, carries a repertoire of at least one anti-sense transcriptional start site on approximately 46 % of its open-reading frames, 28 % of tRNAs, and the 5′ leader sequences for both 16S rRNA and 23S rRNA [3]. Barring a few exceptions, sRNAs are typically 50 to 500 nucleotides in length and do not code for proteins [4–6]. Regulatory functions can predominantly be attributed to their interactions with proteins or target mRNA transcripts [7, 8]. The sRNAs in the latter category manipulate RNA transcription through either cis-acting or trans-acting mechanisms [4, 9]. By definition, cis-acting sRNAs are encoded on the anti-sense strand and generally display perfect nucleotide complementarity with the target sequence in the open reading frame. Trans-acting sRNAs, on the other hand, are encoded within the intergenic regions, act on the targets elsewhere in the genome, and possess short segments of partial nucleotide complementarity to their target genes [6–8]. Accordingly, they require a known RNA chaperone, namely Hfq, encoded by nearly 50 % of all bacterial species to facilitate their binding interactions with an mRNA transcript [2, 7]. In other organisms such as *Listeria monocytogenes*, however, most trans-acting small RNAs function independent of the chaperone activity of Hfq [10].

The genus *Rickettsia* includes obligate, intracellular Gram-negative bacteria belonging to the class *Alphaproteobacteria*. Based on the transmitting natural vector, disease presentation, and antigenicity, this genus was traditionally divided into spotted fever and typhus as two major groups, but sophisticated molecular phylogenetic analysis now classifies rickettsiae into four groups, namely ancestral (*R. bellii* and *R. canadensis*), typhus (*R. prowazekii* and *R. typhi*), transitional (*R. australis, R. akari*, and *R. felis*), and spotted fever (*R. rickettsii*, *R. conorii, R. massiliae*, and numerous more) [11]. Upon transmission into humans from the arthropod vector, vascular endothelial cells are the primary targets of rickettsial infections, with the notable exception of *R. akari*, which primarily invades macrophages [12, 13]. Among well-known human rickettsioses, Rocky Mountain spotted fever (RMSF) due to *R. rickettsii* and epidemic typhus caused by *R. prowazekii* are considered to be the most severe forms of disease. Without proper antibiotic treatment, mortality rate for RMSF is approximately 20 % [14, 15], while the same for epidemic typhus reportedly ranges from 10 % to 60 % [12, 16]. Also, *R. prowazekii* is unique in that patients can harbor sub-clinical infections after successful treatment of the primary infection and later develop recrudescent typhus, also known as Brill-Zinsser disease, despite being symptom-free for years [16, 17]. On the other hand, Mediterranean spotted fever and endemic typhus, caused respectively by *R. conorii* and *R. typhi,* generally represent relatively milder forms of spotted fever and typhus [18, 19].

Due to the historic importance of rickettsial diseases, their global distribution and associated morbidity or mortality, and potential implications in bioterrorism, the genome of *R. prowazekii* was the first to be sequenced and published [20]. Unlike other intracellular bacteria, whose genomes have very high coding densities, *R. prowazekii* was found to have 24 % non-coding DNA [20, 21]. Such large amount of non-coding DNA was projected to be the consequence of genomic reduction and pseudogenization due to the loss or degradation of genes involved in several mechanisms leading to obligate intracellular lifestyle of this pathogen. A number of other rickettsial genomes, including those of *R. rickettsii, R. conorii, R. typhi,* and other notable species have since been sequenced and either published or made available in biomedical databases [22–24]. However, the presence of small, non-coding RNAs in different *Rickettsia* species still remains undetermined. With an aim to address this important knowledge gap, we used SIPHT/sRNAPredict2 to identify candidate novel sRNAs within the intergenic regions of all four rickettsial groups, leading to the prediction of a total of 1,785 novel sRNAs within 16 different strains representing 13 rickettsial species. We further analyzed the predicted sRNAs in *R. prowazekii* strain Brienl using other bioinformatic tools and experimentally validated their expression using reverse transcriptase-polymerase chain reaction (RT-PCR) and deep sequencing approaches. In tandem, these analyses constitute the very first evidence documenting the presence of sRNAs in rickettsial genomes.

## Results

### Bioinformatic prediction of Small RNAs

Ready availability of complete genome sequences render computational approaches a widely acceptable first step for identification of sRNAs [25–27]. Such bioinformatic approaches search the intergenic regions (IGRs) of a bacterial genome for specific sRNA features. In general, the strategy involves an in-depth search of IGRs for the presence of Rho-independent terminators, promoters, and transcription factor binding sites, followed by the analysis of their secondary structure and comparison of such IGRs with closely related species [3, 26, 28]. In this study, we employed the web-based program SIPHT to examine the genomes of 16 rickettsial strains, which represent a total of 13 species spanning all four rickettsial groups. Four known plasmids, representing three from the spotted fever group strains and one from a transitional group strain, were also included. The *R. felis* pRFdelta plasmid (NC_007111.1) was excluded from the analysis as it was found to be an artifact from genome assembly [11]. We primarily employed recommended default settings to identify most sRNA features. To perform a more stringent search, however, we chose to decrease the Expectation Value (E value) from the default 5e^−3^ to 1e^−15^ to minimize false positives. As a result, we identified a total of 1,785 candidate rickettsial sRNAs (Additional file 1). On average, we predicted 74 candidates per ancestral strain, 21 candidates per typhus strain, 152 candidates per transitional strain, and 158 candidates per spotted fever strain. Table 1 categorizes the number of predictions by nucleotide size and rickettsial strain. Of the four plasmids examined, *R. peacockii* plasmid RPR had a single sRNA prediction.

**Table 1:** sRNA predictions categorized by nucleotide size

Predicted sRNA nucleotide size							
Rickettsia	30–100	101–200	201–300	301–400	401–500	500–550	Total
Ancestral group							
* R. bellii OSU*	33	55	6	5	1	0	100
* R. bellii RML*	39	43	13	3	2	0	100
* R. canadensis*	22	14	7	1	1	2	47
Typhus group							
* R. prowazekii Breinl*	8	11	5	2	0	0	26
* R. prowazekii Madrid E*	8	11	5	2	0	0	26
* R. typhi*	4	5	3	2	1	0	15
Transitional group							
* R. akari*	46	43	19	5	1	2	116
* R. felis*	75	80	25	6	2	0	188
Spotted fever group							
* R. rickettsii Iowa*	53	49	13	8	1	1	125
* R. rickettsii Sheila Smith*	56	54	17	8	0	0	135
* R. africae*	62	67	19	10	3	2	163
* R. heilongjiangensis*	54	69	12	9	3	1	148
* R. conorii*	62	59	15	4	3	3	146
* R. japonica*	67	72	22	12	4	1	178
* R. massiliae*	64	66	19	6	2	0	157
* R. peacockii*	79	74	17	9	8	4	191

SIPHT predicted sRNAs categorized by both rickettsial groups and nucleotide size

### Computational analysis of sRNA predictions

Based on the predictive analysis suggesting sRNAs in all rickettsial groups, we set out to analyze each of the candidate sRNAs. In order to examine a common set of bacterial sRNAs within *Rickettsia* species, we first investigated five well-known bacterial sRNAs, namely 6S RNA (*ssrS*), α-tmRNA (*ssrA*), RNaseP_bact_a, rpsL_ricks, and 4.5S RNA (*ffs*), and confirmed their presence in *R. prowazekii*. Using BLAST with an E-value cut-off of 1e^−5^, we compared the prediction for each strain against others included in the study to find shared sRNA candidates [29]. As expected, we noted that rickettsial strains closely related to each other phylogenetically had a greater number of shared sRNA candidates when compared to those that are distantly related (Table 2). For example, *R. rickettsii* strain Sheila Smith (virulent) and strain Iowa (avirulent), which share 96.6 % homology [30, 31], had 115 sRNAs in addition to ten other sRNA predictions in Iowa, and 20 present only in Sheila Smith.

**Table 2:** sRNA comparison

	*R. bellii *OSU	*R. bellii *RML	*R. canadensis*	*R. prowazekii *Madrid	*R. prowazekii *Breinl	*R. typhi*	*R. felis*	*R. akari*	*R. rickettsii *SS	*R. rickettsii *IA	*R. conorii*	*R. africae*	*R. heilongjiangensis*	*R. japonica*	*R. massiliae*	*R. peacocki*
Ancestral																
*R. bellii *OSU	----------	86	5	4	4	4	58	6	20	24	26	19	23	22	14	32
*R. bellii *RML		----------	4	4	4	4	60	7	21	24	33	21	28	25	15	37
*R. canadensis*			----------	3	3	3	11	8	6	5	6	6	5	7	8	3
Typhus																
*R. prowazekii *Madrid E				----------	31	7	5	4	5	3	5	5	3	2	4	2
*R. prowazekii *Breinl					----------	7	5	4	5	3	5	5	3	2	4	2
*R. typhi*						----------	3	3	3	4	3	3	1	1	3	1
Transitional																
*R. felis*							----------	67	85	65	86	90	78	84	86	91
*R. akari*								----------	37	34	42	41	41	36	39	38
Spotted fever																
*R. rickettsii *SS									----------	120	98	103	88	98	87	98
*R. rickettsii *IA										----------	103	106	96	107	94	105
*R. conorii*											----------	126	93	102	94	111
*R. africae*												----------	100	118	104	121
*R. heilongjiangensis*													----------	136	82	98
*R. japonica*														----------	93	109
*R. massiliae*															----------	92
*R. peacocki*																----------

Comparison of sRNA predictions and five well-known bacterial sRNAs (6S RNA, α-tmRNA, rpsL_ricks, 4.5S RNA, RNaseP_bact_a) against other rickettsial species and strains. Shown are the numbers of sRNAs that demonstrate similarity after a BLAST comparison (E-value < 1e^−5^)

We next employed the web-based program, BPROM, to determine promoter motifs for predicted sRNAs in the spotted fever and typhus group of rickettsiae, since they represent the most prominent groups of human pathogens. While it is still undetermined whether the ancestral group causes human disease, the transitional group species are established pathogens, but they account for a small fraction of reported rickettsiosis cases. Using all sRNA predictions within the typhus group (3 strains) and the spotted fever group (8 strains), we searched 150 nucleotides upstream of the predicted sRNA start site for the -10 and -35 promoter motifs because nearly 80 % of known σ^70^ promoters in *E. coli,* considered to be a model organism, fall within 150 bp of the transcription start site [32]. While BPROM successfully predicted the -10 and -35 promoter sites for all candidate typhus group sRNAs, it was unable to predict a promoter site for one sRNA candidate (#132) belonging to *R. rickettsii* strain Sheila Smith.

**Fig. 1:**
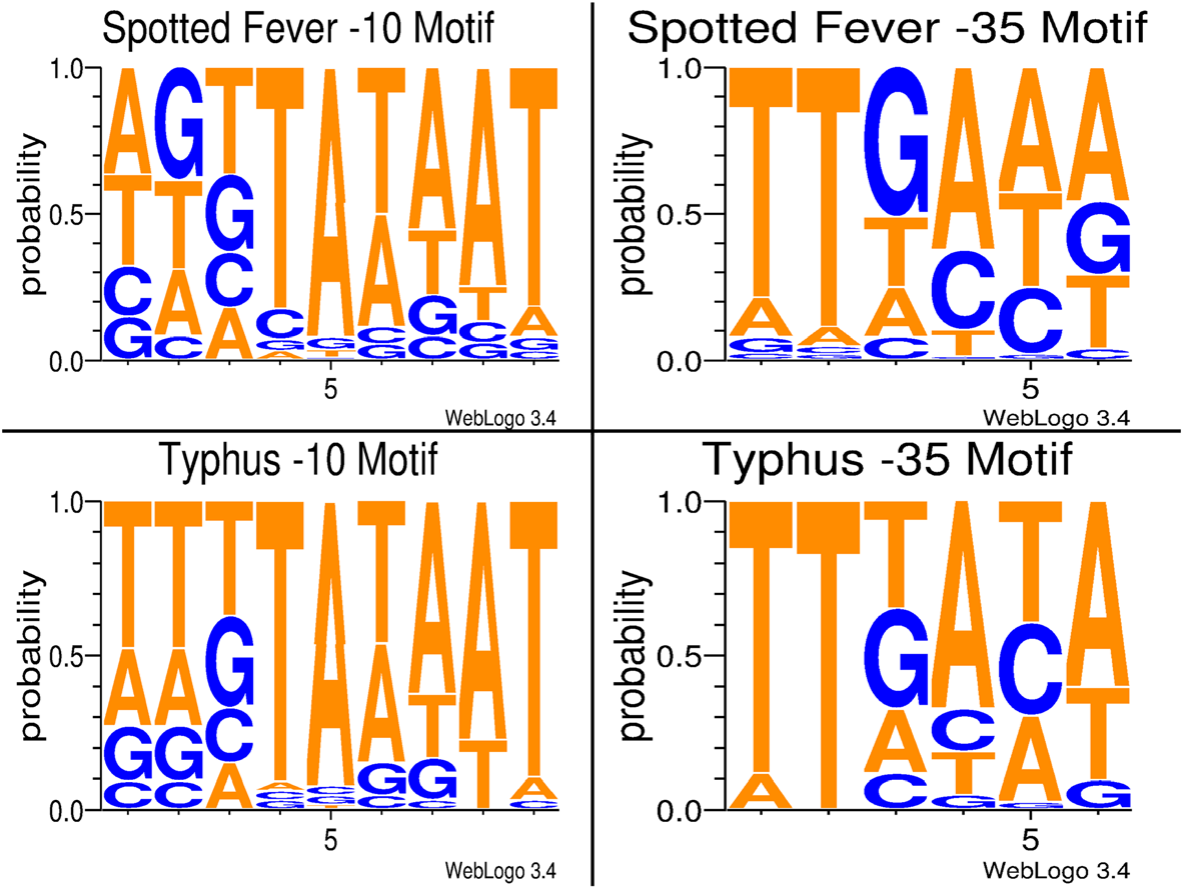
sRNA promoter frequencies. Conservation diagrams illustrating the probability of a nucleotide in a specific promoter motif position. The left side demonstrates the −10 promoter motif, while the right side is the −35 promoter motif. The upper portion displays the typhus group, while the lower displays the spotted fever group. Both groups have −10 motifs similar to the *E. coli* consensus sequence (TATAAT). On the other hand, the −35 motifs vary when compared to the *E. coli* consensus sequence (TTGACA)

Using the data obtained from the BPROM software, we calculated the average distance for the predicted -10 motif and -35 motif for both spotted fever and typhus groups of rickettsiae. For the typhus group, -10 and -35 motifs were an average of 67 and 88 (Stan. Dev. = ±22) nucleotides upstream of the sRNA start site, respectively. The spotted fever group had similar nucleotide distances at 70 and 91 (Stan. Dev = ±22) nucleotides upstream. The average distance between the -10 and -35 motifs was 21 nucleotides for both groups, slightly longer than the reported 17 ± 1 nucleotide distance optimal for *Escherichia coli* genes [33, 34]. Typhus group and spotted fever nucleotide frequencies for the -10 and -35 motifs were plotted using WebLogo3 (Fig. 1). The known consensus sequences of *E. coli* open reading frames (ORFs) for the -10 motif and -35 motif are TATAAT (with each nucleotide probability at 82 %, 89 %, 52 %, 59 %, 49 %, 89 %) and TTGACA (with each nucleotide probability at 69 %, 79 %, 61 %, 56 %, 54 %, 54 %), respectively [34]. Both rickettsial groups favored a -10 motif similar to the consensus sequence. Interestingly, the −35 motif differed between the groups, as well as from the *E. coli* consensus sequence, at the fifth nucleotide. In *E. coli*, this position is cytosine in 54 % of tested sequences. However, it is adenosine (approximately 41 %) or thymine (approximately 40 %) in spotted fever and thymine (approximately 35 %) or adenosine (approximately 30 %) in typhus. In addition, the second nucleotide position in typhus is most conserved with a thymine in nearly 100 % of predicted sites, while it is approximately 90 % for spotted fever. This is in contrast to *E. coli*, which has a thymine with 79 % probability at the same nucleotide position.

**Fig. 2:**
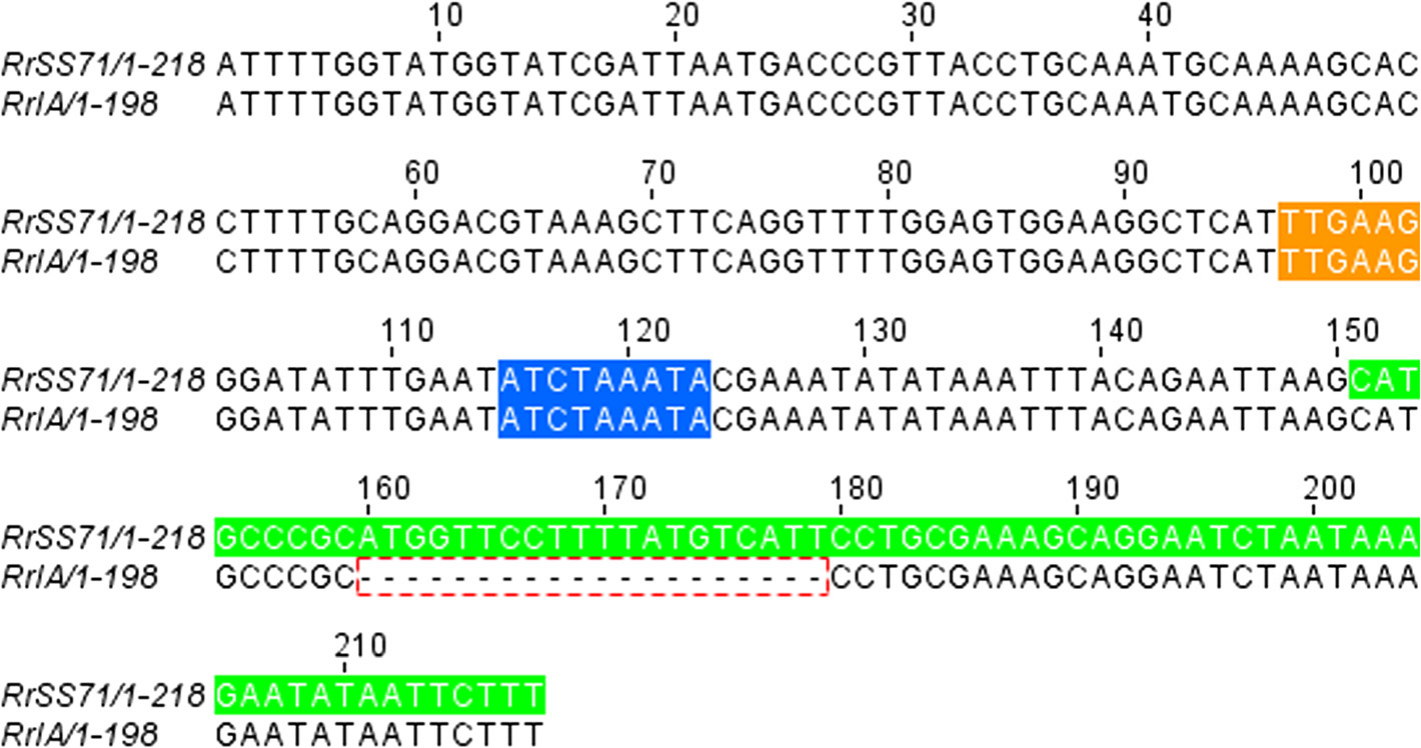
Alignment of *R. rickettsii* strain Sheila Smith sRNA candidate #71. The sRNA candidate #71, predicted only in R. *rickettsii* strain Sheila Smith but not in strain Iowa and the upstream 150 bp region of predicted sRNA were aligned with the corresponding genomic region from strain Iowa. The predicted −10 box (orange), −35 box (blue), and sRNA sequence (green) are highlighted. A 20bp deletion observed in the genomic sequence of strain Iowa is shown by the dotted line

**Fig. 3:**
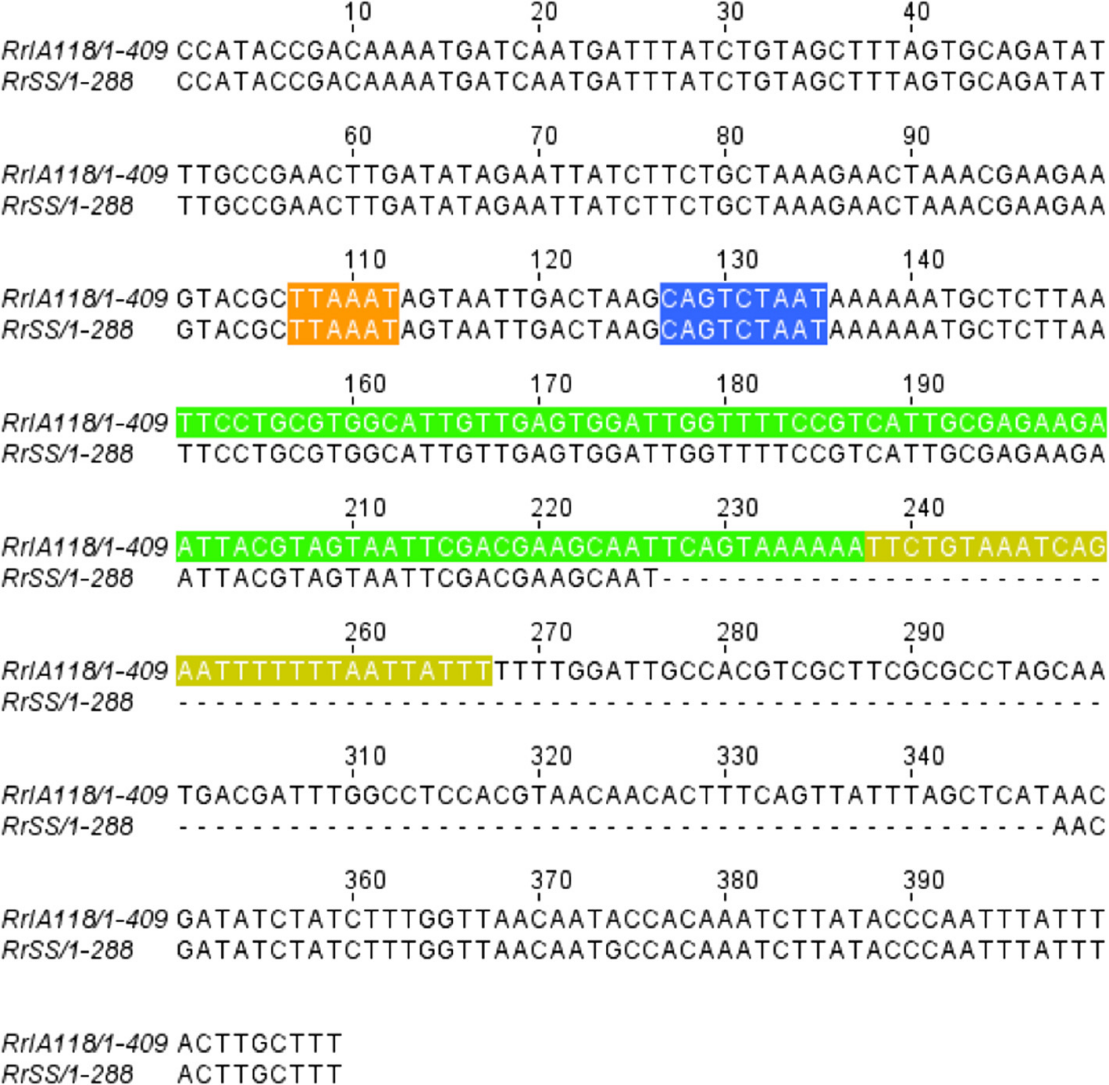
Alignment of *R. rickettsii* strain Iowa sRNA candidate #118. The sRNA candidate #118, predicted only in R. *rickettsii* strain Iowa but not in strain Sheila Smith and the 150 bp up- and downstream regions of predicted sRNA were aligned with the corresponding genomic region from strain Sheila Smith. The predicted −10 box (orange), −35 box (blue), sRNA sequence (green) and the Rho independent terminator (yellow) are highlighted. A nucleotide sequence absent in the genomic sequence of strain Sheila Smith and mapping to the predicted sRNA and the Rho independent terminator in strain Iowa is shown by the dotted line

In an attempt to explain the differences in sRNA predictions between Sheila Smith and Iowa strains of *R. rickettsii*, we performed a comparative analysis by mapping the sRNAs present only in one of the strain but absent in another. Accordingly, we compared 20 predictions from Sheila Smith strain and their corresponding 150bp up- and downstream sequences to the strain Iowa genome. All “prediction ± 150 bp” sequences were >99 % identical with the exception of two (#23 and #71) sRNAs. For instance, sRNA candidate #71 had a 20 bp sequence absent from the predicted sRNA sequence in the Iowa genome (Fig. 2). The same analysis was conducted for the 10 predictions present only in *R. rickettsii* strain Iowa but absent in strain Sheila Smith. Again, all but two “prediction ± 150 bp” sequences were nearly identical (>99 %). In this case, prediction #118 for strain Iowa had a 46 bp sequence that was absent in strain Sheila Smith (Fig. 3). Also, an ORF was annotated in corresponding genomic regions in strain Sheila Smith for nearly 30 % of sRNAs predicted only in strain Iowa, while two sRNAs had SNPs and indels in the Sheila Smith sequences potentially leading to altered thermostability of secondary structures. Similar observations were made for sRNAs present only on Sheila Smith but absent in Iowa strain. Since SIPHT relies on the conserved intergenic regions, secondary structures, presence of promoters, and terminator sequences, it is likely that the sRNAs predicted only in one strain, but not the other, result from these stringent criteria.

### Candidate sRNA target identification

Although spotted fever rickettsiae cause disease worldwide, we chose to initially focus on *R. prowazekii* due to the high public health threat. In addition, due to bioweapon testing during World War II and the development of antibiotic resistant strains during the Cold War, *R. prowazekii* remains on the list of select agents with potential for bioterrorism [35]. To identify potential mRNA targets for predicted sRNAs within the *R. prowazekii* genome, we chose two independent web-based programs, TargetRNA2 and CopraRNA, to predict sRNA:mRNA interactions by assessing the base pairing potential based on a Smith-Waterman dynamic and conservation profile, respectively [36–38]. The search parameters were set to default and a p-value threshold of ≤0.05. A total of 393 potential targets were identified by TargetRNA2, whereas CopraRNA predicted 1154 protein coding genes to be regulated by sRNAs. A detailed comparative analysis revealed that 16 sRNA candidates had common target genes predicted by both programs and the remaining 10 candidates had independent predictions (Additional file 2). Two sRNA candidates (#6 and #22) had the highest number of 6 common targets predicted by TargetRNA2 and CopraRNA, in contrast to only one commonly predicted target for candidates #12 and #19. In summary, a total of 51 target genes were predicted by both programs, of which only 9 were categorized as hypothetical proteins. Of note, target genes such as *virB10, ftsL, ftsQ, secA, ruvB,* and 190kDa antigen were commonly predicted, indicating the potential role of post-transcriptional regulatory mechanisms in type IV secretion, cell division, and DNA repair. Table 3 lists the number of predicted target transcripts for each predicted sRNA. Interestingly, TargetRNA2 failed to predict targets for candidate #7, but CopraRNA predicted 53 target genes (Table 3). Nevertheless, if the lack of target prediction by TargetRNA2 holds true for candidate #7, this may be due to a sRNA:protein interaction, as is the case with 6S RNA, or it may simply represent a degraded ORF with an active promoter and terminator. Using this information, we parsed the predicted mRNA targets based on their respective protein function. We selected eight categories, including a category for ‘other’ (annotated ORFs with known function, but not categorized into a separate class based on function) and ‘hypothetical proteins’ (annotated ORFs with unknown/uncharacterized function), and separated the 393 predictions into different categories. The categories included cell division, cell wall, metabolism, ribosomal functions, virulence, type IV secretion system, transport proteins, and phagosomal escape (Table 4). Our TargetRNA2 and CopraRNA results demonstrate that the majority of known targets for sRNAs are involved in metabolism (71 vs. 197), ribosomal functions (51 vs. 129), and cell division (44 vs. 30). However, both the programs predicted a large number of target genes potentially regulated by sRNAs that were categorized as ‘other’ (90 vs. 370) and ‘hypothetical proteins’ (73 vs. 232), respectively (Table 4). While TargetRNA2 uses Smith-Waterman dynamic based base pairing, CopraRNA predictions are largely based on conservation between different genomes, possibly resulting in the differences in the number of predicted targets.

**Table 3:** sRNA target predictions

sRNA prediction	Number of targets predicted by	
	TargetRNA	CopraRNA
1*	39	47
2	3	43
3	4	40
4	11	23
5*	27	50
6	21	49
7	0	53
8	8	37
9*	19	43
10*	32	49
11	17	49
12	11	44
13	17	49
14	13	37
15	25	50
16	12	45
17	18	51
18	2	54
19	9	45
20	10	39
21	12	39
22	14	46
23	10	45
24*	24	54
25*	29	36
27	6	37
Total	393	1154

Number of target predictions per each sRNA for *R. prowazekii* strain Breinl. Candidate #26 is not listed, as SIPHT provided no #26 prediction. Asterisks represent those candidates selected for confirmation of expression

**Table 4:** sRNA target categorization

Target classification	Number of predicted targets by category	
	TargetRNA2	CopraRNA
Cell division	44	30
Cell wall	24	83
Metabolism	71	197
Ribosomal protein	51	129
Virulence	3	17
T4SS	2	28
Other	90	370
Transport	33	67
Phagosome escape	2	1
Hypothetical protein	73	232
Total	393	1154

Target genes are classified into ten categories based on either known or hypothetical function for *R. prowazekii* strain Breinl

### RNA sequencing

Next, we set out to confirm the expression of rickettsial sRNAs during infection of cultured human microvascular endothelial cells (HMECs) with *R. prowazekii*. We infected HMECs with *R. prowazekii* strain Breinl and extracted total RNA at 3 and 24 h post-infection. Our rationale for choosing these durations was to allow ample time for rickettsial entry and establishment of infection within the host cells and sufficient time for at least two replication cycles keeping in mind that replication time for intracellular rickettsiae ranges from 9 to 11h. After removal of rRNAs and eukaryotic mRNA, the enriched bacterial RNA was reverse transcribed into cDNA libraries and subjected to next generation sequencing using the Illumina HiSeq™ 1500 system. The resulting RNA reads were mapped onto the *R. prowazekii* strain Breinl (NC_020993) genome. Our deep sequencing resulted in approximately 42.6 to 46.2 million total reads for RNA isolated at 3 h and 27.4 to 28.6 million total reads at 24 h post-infection. Out of these, an average of 1.4 and 2.8 million reads mapped to the *R. prowazekii* genome at 3 h and 24 h, respectively. *Rickettsia* species include obligate intracellular bacteria with fastidious growth requirements in a host cell and cannot yet be cultured in a cell-free environment. Recently, it has been reported that intracellular organisms such as *Rickettsia* represent only 5 % of the extracted total RNA, while the remaining 95 % belongs to the eukaryotic host. Out of approximately 5 % bacterial total RNA, 95 % is composed of ribosomal and transfer RNA, while the remaining 5 % of the transcripts correspond to bacterial mRNA and sRNA, yielding a ratio of ~1:400 bacterial mRNA and sRNA in total RNA extracted during the infection [39]. Although microbe enrichment is aimed at removing most of the polyadenylated eukaryotic transcripts and ribosomal RNAs, the process often accomplishes only limited removal of other interfering eukaryotic RNAs such tRNAs, noncoding RNAs, and mitochondrial RNA. Furthermore, high abundance of rRNAs in the host cells also interferes with the efficacy of their removal from the sample preparations. Supporting our results, a recent study has reported that only 2–5 % of the total reads mapped to the intracellular bacterial genomes despite enrichment of the total RNA [39]. By analyzing the sequencing data at the genome locations predicted by SIPHT, we found that twelve out of 26 predicted sRNA had a Mean Expression Value (MEV) that was ≥1.5 times compared to their respective 50 nucleotide upstream and downstream flanking regions (Table 5). As expected, all five well known sRNAs, namely 6S RNA, α-tmRNA, RNaseP_bact_a, rpsL_ricks, and 4.5S RNA were found to be expressed in vitro and exhibited an MEV of >1.5. The read coverage plots of 6S RNA, RNaseP_bact_a and α-tmRNA are presented in Additional file 3.

**Table 5:** sRNA predicted promoter locations

Candidate	Start position	Stop position	Strand	−10 box	−35 box
2	163641	163556	Anti-sense	ATCTAGGAT	TTAATT
5	659164	659057	Anti-sense	TTGTATTAT	TTTATT
6	644329	644199	Anti-sense	TAGTAAAAA	TTAGAA
9	457001	456876	Sense	ACTTATCAT	TTGCTG
10	371859	371506	Anti-sense	TGTTAAAAT	TTTATT
11	308324	308042	Anti-sense	TTTTGAAAT	TTCTAA
12	306070	305924	Sense	ATGTATATT	TTGATG
21	47692	47542	Sense	GGGTATAAC	ATGACA
22	10482	10278	Anti-sense	ATGTAAGAT	TTTACT
23	1105018	1104959	Sense	GATCAGAAT	TTCAAA
24	1039473	1039278	Anti-sense	ATGTAGATT	TTGATT
25	998167	997927	Anti-sense	GACTAAAAT	TTGCCA

This table outlines those *R. prowazekii* strain Breinl sRNA predictions that had an MEV ≥1.5. It includes the SIPHT predicted start and stop positions as well as the predicted strand. In addition, it contains the BPROM predicted −10 box and −35 box for the σ^70^ promoters

### Validation of sRNA predictions via RT-PCR

**Fig. 4:**
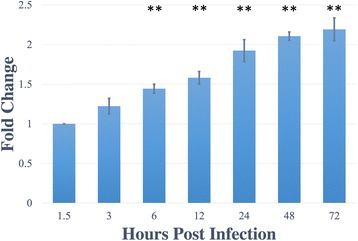
6S RNA (*ssrS*) expression during host cell infection. *R. prowazekii* strain Brienl 6S RNA (*ssrS*) expression was measured during the infection of HMECs over a course of 72 h (*n* = 5). The expression was normalized to 16S rRNA (endogenous control) and baselined to 1.5h post infection. Significant increase was observed starting at 6h post infection. Data is represented as Mean ± SEM. **p **<** 0.01

**Fig. 5:**
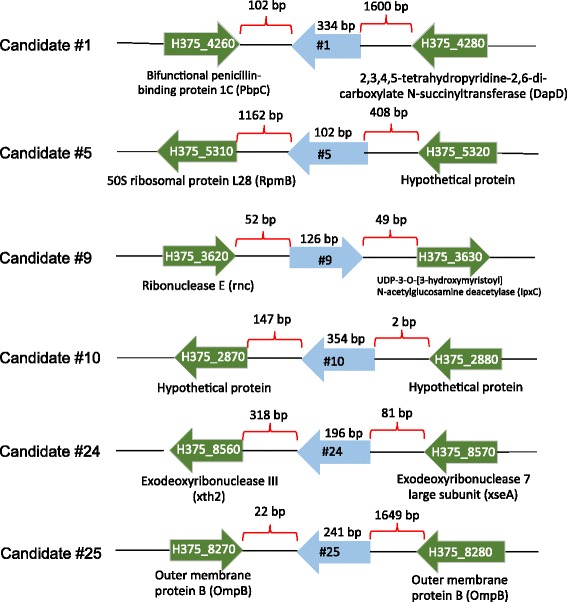
Genomic location of *R. prowazekii* strain Brienl sRNAs. Schematic representation of sRNAs identified to be expressed in *R. prowazekii* strain Brienl during the infection of HMECs. Green arrows represent the orientation of flanking ORFs in relation to the sRNA depicted by blue arrows. The nucleotide distance between the sRNA and the flanking ORF is shown above the brace

Prior to validating our predicted sRNAs, we decided to investigate the expression of 6S RNA within *R. prowazekii.* The underlying rationale for choosing 6S RNA to begin with was its particularly high abundance in *E. coli,* which can reach ~10,000 copies during late stationary phase [40]. Using 16S rRNA as the endogenous control and infection for 1.5 h as baseline, we demonstrated a significant (*p* < 0.01) increase in its expression from 6 to 72 h post-infection (*n* = 5) using TaqMan-based real-time RT-PCR (Fig. 4). After confirming expression of 6S RNA, we chose nine sRNA candidates (#1, #2, #5, #9, #10, #11, #21 #24, and #25) to verify their expression in *R. prowazekii* str. Brienl during infection of HMECs. These were chosen based on their location within the genome, orientation comparative to the neighboring genes, and potential mRNA targets (Fig. 5). Candidates #11 and #21 were not detected using RT-PCR. However, the remaining seven candidates were detected using RT-PCR (*n* = 3) with an amplicon near the expected size.

**Fig. 6:**
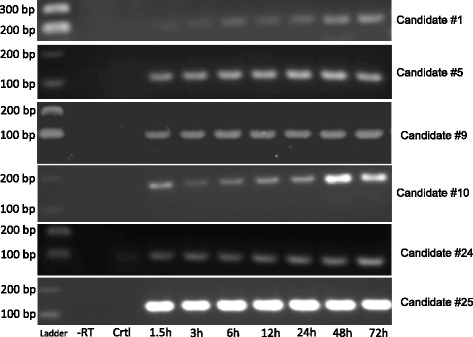
Expression of *R. prowazekii* strain Brienl candidate sRNAs during host cell infection. The *R. prowazekii* strain Brienl sRNA candidates #1,#5, #9, #10, #24, and #25 were tested for their expression during infection of HMEC by RT-PCR (*n* = 3). The band sizes shown on the left side correspond to the 100 bp DNA ladder (New England Biolabs). The lane 2 (-RT) is a “no reverse transcriptase” control, while the lane 3 (Crtl) is an uninfected HMEC control. Lanes 4 through 10 are the samples from *R. prowazekii* strain Brienl infected HMECs from 1.5h to 72h post infection. All the tested sRNA candidates showed expression during host cell infection

Figure 6 shows a representative agarose gel for candidates #1, #5, #9, #10, #24, and #25. Upon cross-reference with other *R. prowazekii* genomes that included strains Madrid E, Dachau, BuV67, Katsinyian, Chernikova, RpGvF24, GvV257, and Rp22, it was found that candidate #2 was anti-sense to the gene *rnpB* (RNaseP_bact_a) annotated only in *R. prowazekii* strain Rp22 (NC_017560). Therefore, any amplification is likely the result of *rnpB* expression. The remaining predictions demonstrated no association with any other annotated open-reading frames. To further confirm this observation, each sRNA sequence was examined for its ability to code for a protein. Using the ExPASy Translate Tool (Swiss Institute of Bioinformatics), all six possible translation initiation positions (3 each on 5′ and 3′ strands) were assessed to be devoid of protein coding capacity, yielding evidence that these are indeed small non-coding RNAs expressed during rickettsial infection of host endothelium.

## Discussion

In this study, we report on a genome wide computational analysis to identify novel sRNAs within the genus *Rickettsia.* We have identified 1,785 sRNAs in 16 rickettsial strains belonging to 13 different species and spanning across all rickettsial groups. To further confirm our sRNA predictions, we have validated the expression of six predicted trans-acting sRNAs in *R. prowazekii* strain Breinl using high throughput sequencing and RT-PCR approaches. Since the initial discovery of sRNAs in 1960s, *E. coli* has been shown to harbor nearly 80 to 100 small RNAs, while *Salmonella enterica* serovar Typhimurium genome encodes for ~140 small RNAs [4, 41, 42]. Abundant evidence now demonstrates the ubiquitous nature of sRNAs in bacterial genomes and implicates them to play an important role in virulence, quorum sensing, survival, plasmid expression, and primary and secondary metabolism in addition to several other housekeeping functions [43–49]. In *Vibrio cholerae* and *V. harveyi,* quorum-sensing genes *hapR* and *luxR* are under the regulatory control of four and five sRNAs, respectively [46]. Furthermore, the deletion of three sRNAs in *Listeria monocytogenes* results in an attenuated phenotype in mouse models and the mutant strain is unable to grow in murine macrophages [44]. Similarly, *rli38* knockout mutants of *Listeria* were found to be attenuated in orally infected mice, suggesting a role in the pathogen’s virulence [50], and the deletion of *lhrA* in *L. monocytogenes* was capable of altering the expression of over 300 genes [10]. In *Salmonella enterica,* the AmgR small RNA controls the expression of the *mgtCBR* mRNA required for survival in macrophages and its over expression leads to decreased virulence in mouse models [45]. However, studies examining the potential regulatory roles of sRNAs in obligate intracellular bacteria remain rather limited. In addition to an sRNA that regulates *hctA*, 16 trans-acting and 25 cis-acting sRNAs have been identified in *Chlamydia trachomatis*, an intracellular human pathogen [51, 52]. More recently, *Coxiella burnetii* and *Buchnera aphidicola* genomes are shown to encode for 14 and 140 sRNAs, respectively, and *Coxiella* sRNAs exhibit differential expression at different growth stages [53–55]. Here, we report on the existence of novel small RNAs in rickettsial genomes and their potential roles as determinants of pathogen virulence, host adaptation, and metabolism.

Several prediction programs using parameters such as comparative genomics, RNA structure, and thermodynamic stability, have been developed and utilized to identify bacterial small RNAs [37, 56–59]. In this study, we have chosen SIPHT to predict trans-acting sRNAs in rickettsial genomes as this prediction tool uses several other well established and widely used programs to identify potential transcription factor binding sites [60–62]; Rho-independent terminators using RNAMotif [63], TransTermHP [64], and FindTerm [65]; conserved secondary structures by QRNA [56]; and conserved nucleotide sequences by BLASTN 2.0 [60]. Further, this program has been widely applied for sRNA predictions in several other bacteria attesting to its potential for accurately predicting bacterial sRNAs and its web-based availability makes it both user-friendly and easily accessible. Additionally, unlike its counterparts such as eQRNA and RNAz, SIPHT specifically searches for Rho-independent terminators and conserved intergenic structures significantly eliminating the chances of false-positive predictions [27]. Using SIPHT, we have predicted an average of 21, 74, 152 and 158 sRNAs in typhus, ancestral, transitional, and spotted fever groups of *Rickettsia* species, respectively. To test if predicted sRNAs have upstream transcription factor binding sites and downstream Rho-independent terminator (two independent criteria used by SIPHT), we have further analyzed all *R. prowazekii* sRNAs using BPROM [66] and TransTermHP [64]. All *R. prowazekii* sRNAs have a predicted upstream σ^70^ promoter and a Rho-independent termination confirming the results retrieved from SIPHT (Fig. 1).

Our genus-wide global analysis suggests that the repertoire of predicted sRNAs is independent of the size of respective rickettsial genomes. This is exemplified by the presence of 191 sRNAs in 1.31Mbp genome of *R. peacocki* (spotted fever), in contrast to only 100 sRNAs in 1.54Mbp genome of ancestral *R. bellii*. The number of sRNAs among rickettsiae in different groups, however, tends to directly correlate with their respective genome size, while the average number of sRNAs per Mbp of the genome within a particular group varies depending on the *Rickettsia* species/strain. For example, *R. bellii* and *R. canadensis,* belonging to the ancestral group and carrying the genomes of 1.54Mbp and 1.15Mbp, respectively, encode for 100 and 47 sRNAs. On the other hand, *R. canadensis* has only 40 sRNAs/Mbp, while *R. bellii* has 65 sRNAs/Mbp, indicating the impact of genomic content and organization on the prediction of sRNAs. Also, although the average length of sRNAs in *R. bellii* and *R. canadensis* are fairly similar (132 *vs* 149), a detailed analysis of the length of intergenic regions (IGRs) in *R. bellii* and *R. canadensis* revealed that *R. bellii* has ~63 % more IGRs ranging from 1–300 bp. It is, therefore, possible that the lower number of sRNAs in *R. canadensis* is due to the differences in the number of IGRs included in the SIPHT analysis. Another notable difference is evident between *R. akari* and *R. felis* in the transitional group. While both had over 100 predictions, the coding density of sRNAs in *R. akari* (94 sRNAs/Mbp of genome) was 22 % lower in comparison to *R. felis* (121 sRNAs/Mbp of genome). *Rickettsia* are generally presumed to exhibit limited horizontal gene transfer (HGT) due to their obligate intracellular life-style. However, recent reports document the dynamic nature of their genomes and transposable elements, palindromic repeats, and horizontally acquired genes have been identified in several *Rickettsia* species [11, 67, 68]. For example, the transposable elements in *R. felis* cause inactivation of genes or integration of foreign DNA resulting in changes to both genomic content and arrangement [69]. In this regard, at least 79 genes in *R. felis* have been suggested to be acquired through horizontal gene transfer from other proteobacteria or amoebae [70]. Additionally, *R. prowazekii* and *R. typhi*, despite having similar size genomes, encode for 26 and 15 sRNAs, respectively. Again, the number of IGRs included in the SIPHT analysis varies between *R. prowazekii* strain Brienl and *R. typhi* strain Wilmington (540 vs 504), which may explain the differences in the total number of predicted sRNAs in these typhus rickettsiae genomes.

Although computational approaches yield convincing evidence for the existence of sRNAs in *Rickettsia*, it is critical to validate the expression of predicted sRNAs during host-pathogen interactions via experimental strategies. In this context, we first attempted to confirm the expression of 6S RNA (*ssrS*), a well-characterized small, noncoding RNA ubiquitously present in most bacterial lineages, including *Gammaproteobacteria* and *Bacillales* [53, 71]. Although most bacteria encode a single copy of the *ssrS* gene, some bacterial species including *Bacillus subtilis* reportedly encode for two copies that are differentially expressed depending on the stage of growth [1]. 6S RNA is most abundantly expressed during late stationary phase, where it interacts with RNA polymerase and regulates σ^70^ function. These data further support possible roles of 6S RNA in long-term survival and nutrient uptake [72, 73]. Also, 6S RNA is potentially involved in intracellular stress response in *C. burnetii* and *Legionella pneumophila* [53, 74]. We report a significant increase in the 6S RNA expression from 6 to 72 h post-infection when compared to the basal expression level at 1.5 h. Also, based on our MEV calculations, we observed an increase of 2- and 5-fold in the expression of 6S RNA at 3 and 24 h post-infection, respectively. This is in agreement with earlier findings from other pathogenic bacteria. Specifically, a 2-fold up-regulation in its expression at 72 h appears to correspond to a similar increase seen in *C. burnetii* [53]. Our data further suggest that the highest expression at 72 h post-infection coincides with the intracellular growth kinetics of rickettsiae [75]. A comprehensive analysis to elucidate its mechanisms of action and regulatory functions in intracellular rickettsiae is warranted and currently in progress.

Because RNA sequencing is a novel and robust methodology, which provides valuable insights into the global transcriptome [76], we subjected total RNA from *R. prowazekii*-infected endothelial cells to validate the presence of sRNAs and their expression during host-pathogen interactions. Since the major focus of this study was the identification and validation of intergenic trans-acting sRNAs, additional information on the cis-acting expressed by *R. prowazekii* strain Breinl during infection of HMECs was excluded to perform a direct comparative and confirmatory analysis of SIPHT based sRNA predictions *versus* their expression *in vitro*. Furthermore, none of the web based sRNA prediction tools have the ability to identify cis-acting sRNAs in the genomes. MEV based identification of sRNAs in bacterial genomes is a widely utilized approach that exploits the expression profile of sRNAs and their respective flanking regions to determine the biogenesis of sRNAs [53, 77, 78]. To this end, the reads mapping to each nucleotide of the sRNAs and their respective 50 bp flanking regions were normalized using the total number of reads mapping to the rickettsial genome (excluding those mapping to the rRNAs and tRNAs) and then to their length, and MEVs were determined to decipher the expression of sRNAs from potential read-throughs attributed to flanking ORFs due to leaky transcriptional termination in *R. prowazekii* [79]. Nearly 50 % of predicted sRNAs in *R. prowazekii* exhibited an MEV of ≥1.5 when compared to respective flanking regions indicating their biogenesis and expression independent of neighboring genes. The reads’ coverage plots for 6S RNA, RNAseP_bact_a, and α-tmRNA clearly demonstrate the independent expression of these sRNAs (Additional file 3).

During invasion of epithelial cells and intracellular replication within macrophages, *Salmonella* expresses IsrM RNA encoded in *Salmonella* pathogenicity islands [80]. *L. monocytogenes* encodes a thermosensor sRNA, which upon encountering human body temperature (37 °C) forms an alternative secondary structure and activates adhesins, phagosome escape mechanisms, and other immune-regulating factors [81]. Further assessment of the expression profile of five sRNAs (#5, 9, 10, 24, & 25) showing an MEV of ≥1.5 revealed their steady-state expression of these sRNAs from 1.5 to 72 h post-infection indicating potential roles in pathogenesis. Interestingly, despite an MEV of <1.5, candidate #1 was expressed between 6 to 72 h and its expression increased over time, most notably after 24 h. Since our in-depth transcriptome analysis was performed at 3 and 24 h post-infection, we hypothesize that the relative expression of candidate #1 is likely inadequate to generate sufficient reads to achieve an MEV greater than 1.5 fold. Alternatively, the sequencing depth may not have been high enough to achieve a 1.5-fold difference. Even though 50 % of the predicted sRNAs were either not detected or expressed below the cut-off MEV in our RNA-seq analysis, it is plausible that they are bonafide sRNAs conditionally expressed during other conditions such as stress and host-vector interactions. Previous studies have shown that different environments induce specific sRNAs. Small RNA ryhB, known to down-regulate genes involved in iron storage in *E. coli*, is induced mainly during low iron conditions [82]. In *Salmonella enterica* serovar Typhimurium, IsrJ sRNA is induced under low oxygen and magnesium environments and elevated levels of IsrE are observed in iron responsive environment [83]. Similarly, *H. pylori* is known to induce the expression of six small RNAs (IsoA1-6) associated with acid stress [3]. Alternatively, it is also plausible that SIPHT may have identified degraded ORFs [20, 84]. Rickettsial genomes are known to evolve by reductive evolution (gene degradation) and transposons are known to play a pivotal role in gene inactivation [11, 22, 68, 85, 86]. *R. prowazekii* is known to have pseudogenes potentially resulting from gene inactivation [20]. Since SIPHT uses the presence of an upstream promoter and downstream transcriptional terminator as the main criteria for predicting sRNAs, it is possible that some of sRNA transcripts predicted by SIPHT potentially map to the degrading ORFs, which still retain conserved promoter and terminator regions.

Despite the abundance of sRNAs in all bacterial lineages, little is known about their function and mechanism of action within the bacterial genomes and only a few sRNAs have been assigned with functions till date [53]. Using TargetRNA2 and CopraRNA, we have predicted the target mRNAs regulated by *R. prowazekii* sRNAs. Functional categorization of the target genes regulated by sRNAs resulted in identification of genes involved in key pathways of cell division, transport, phagosomal escape, virulence, type IV secretion system, and metabolism. A majority of these pathways are critical for the growth and survival of *Rickettsia* in the host cytoplasm. For example, we have identified 33 genes involved in transport mechanisms and potentially regulated by sRNAs, a function important for rickettsial survival *in vivo* as they encode for translocases required for the exchange of ADP with ATP from host cell cytosol [11, 87, 88]. Following invasion into host cell, rickettsiae quickly escape escape into the host cytosol by phagosome degradation and published studies have implicated a role of rickettsial hemolysin C (tlyC) and phospholipase D (pld) in phagosomal escape [89, 90]. We have identified two sRNAs, #24 and 27, with the potential to regulate tlyC and pld, respectively, suggesting an important role for these sRNA in the establishment of infection. A significant number (18 %) of predicted target genes were categorized as ‘hypothetical proteins’, which is not surprising considering that nearly 26 % of the 914 *R. prowazekii* genes are still reported as uncharacterized ORFs. As rickettsial genes are further investigated for their functional roles, we anticipate that most of these hypothetical proteins will likely be assigned a role in virulence, survival, and pathogenesis during host-pathogen and vector-pathogen interactions.

## Conclusions

Bacterial small RNAs are now well appreciated as the major post-transcriptional regulators involved in key processes such as virulence, quorum sensing, survival, plasmid expression, and primary and secondary metabolism. *Rickettsia* species, despite undergoing reductive evolution, generally harbor ~25–30 % intergenic regions and presumably encode for trans-acting sRNAs. This study was aimed at identifying trans-acting sRNAs in rickettsial genomes and their possible roles in host-pathogen interactions. We have identified 1785 sRNAs in 13 rickettsial species spanning across all four rickettsial groups, and validated the expression of *R. prowazekii* sRNAs by RT-PCR and high throughput transcriptome analysis. Furthermore, using Taqman assay, we have quantified the expression of *R. prowazekii* 6S RNA, a small RNA known to regulate the housekeeping transcription factor σ70, during host cell infection. Our study is the first to report on the existence of small RNAs in the genus *Rickettsia*. Further studies are required to validate candidate trans-acting and identify cis-acting sRNAs as well as determine their functions in rickettsial physiology and pathogenesis.

## Methods

### Rickettsia species and strains

For this study, available genome sequences of 16 rickettsial strains, encompassing 13 species belong to the genus *Rickettsia* were used. Included species represent all four, namely ancestral, typhus, transitional, and spotted fever, rickettsial groups (Additional file 4). Further, four known rickettsial plasmids were also subjected to the proposed analysis (Additional file 4).

### Prediction of sRNAs

We used the web-based program SIPHT available from the University of Wisconsin at Madison (http://newbio.cs.wisc.edu/sRNA/index.php) for predicting sRNAs in rickettsial genomes [26]. This program predicts sRNAs within the intergenic regions of bacterial genomes by searching for Rho-independent terminators downstream of conserved sequences, followed by an analysis of conservation with other species, potential transcription factor binding sites, the spacing between flanking genes, and homology with known sRNAs [26]. The specific parameters used for all searches were as follows. The maximum expected value for BLAST (BLAST E) was changed from the default setting of 5e-3 to a more stringent value of 5e-15 in order to eliminate the possibility of false positives. The minimum score for BLAST (BLAST S) and minimum percent identity (BLAST % identity) were set at the default 0. The maximum BLAST high-scoring segment pairs (HSP) length was set at 1000. The maximum Rho-independent terminator criteria were 86, -10, and -6 for TransTerm, FindTerm, and RNAMotif, respectively. The minimum predicted locus length was 30, while the maximum predicted locus length was 550. These scores take into account the generally defined 50 to 500-nucleotide length of bacterial sRNAs [4]. The minimum distance by default for both the locus start site to ORF start site and for the locus start site to ORF end site was −65. However, the minimum distance set by default from the locus end site to ORF start site was −20. The minimum distance from the locus end site to ORF stop site was left at 35. Lastly, the minimum distance from the transcription factor-binding site to the ORF start site was 0. This value allows for the inclusion of all possible candidate sRNAs in the final report. To ensure consistency, all of these parameters were set as the default analytical criteria for the program.

### Promoter prediction

For bacterial promoter predictions, the web-based software BPROM was used. This program searches for bacterial σ^70^-family promoter -10 box and −35 box, transcription start site, and other transcription factor binding sites in a given genomic sequence with a reported accuracy and specificity of 80 % [66]. Each promoter prediction was conducted using 150 base pairs upstream of the predicted sRNA start site. Nucleotide frequency plots were created using the −10 box and −35 box predictions. The web based program WebLogo3 from the University of California at Berkeley was used to generate the sequence logos [91].

### Target prediction

Target genes for each candidate sRNA were predicted using the web based program TargetRNA2 [36]. This program searches a genome’s annotated features for a statistically significant base pair-binding potential to the queried nucleotide input and calculates a hybridization score followed by a statistical significance of each potential RNA-RNA interaction [36]. The individual base pair model was used throughout our target prediction procedures. The following parameters were used for each prediction. For statistical significance, the p-value was set at ≤0.05. The program searched 80 nucleotides before the start codon and 20 nucleotides after the start codon. The selected seed length was 7 consecutive nucleotides, which corresponds to the average seed length (6 to 8 nucleotides) for trans-acting sRNAs [4]. The filter size, which corresponds to how the program filters out non-target mRNA, was set at the default 400.

### Cell culture, infection, and RNA isolation

Human dermal microvascular endothelial cells (HMECs) were cultured in MCDB131 medium with L-glutamine (10mmol/L), mouse epidermal growth factor (10ng/ml), hydrocortisone (1μg/mL), and 10 % heat-inactivated fetal bovine serum [92]. The use of HMECs as an established cell line for *in vitro* studies was exempt from the review and approval by the Institutional Review Board, but was approved by the Institutional Biosafety Committee at the University of Texas Medical Branch. Cells were grown at 37 °C with 5 % CO_2_ until approximately 80 to 90 % confluency. *Rickettsia prowazekii* strain Breinl was cultivated in Vero cells and purified by differential centrifugation to prepare seed stocks for host cell infection experiments. Titers were estimated by a combination of plaque formation assay and quantitative PCR (qPCR) using primer pair Rp877p-Rp1258n for citrate synthase gene (*gltA*) [92, 93]. Infection was carried out under appropriate Biosafety Level 3 conditions using approximately 6 X 10^4^ pfu of rickettsiae/cm^2^ of culture surface area. These conditions yield an infection of >80 % of cells with approximately six intracellular rickettsiae per cell [92, 94, 95]. After 15 min of incubation with gentle rocking to allow for sufficient adhesion and invasion, the medium was removed and replaced with fresh medium. Infected cells were then further incubated at 37 °C with 5 % CO_2._ Total RNA was isolated at 1.5, 3, 6, 12, 24, 48, and 72 h post-infection using Tri-Reagent^®^ (Molecular Research Center). RNA was extracted using the Direct-zol™ RNA MiniPrep kit (Zymo Research). Column DNaseI treatment (Zymo Research) was performed on all RNA samples to eliminate contaminating genomic DNA.

### RNA sequencing

In order to sequence the rickettsial transcriptome, HMECs were infected with *R. prowazekii* strain Breinl and total RNA was isolated at 3 and 24 h post-infection using Tri-Reagent (Molecular Research Center). Samples were treated with DNaseI (Zymo Research) to eliminate contaminating genomic DNA and subjected to the MICROBEnrich Kit (Ambion) to remove interfering eukaryotic mRNAs. Ribosomal RNA was then removed using the Ribo-Zero kit (Epicentre). RNA was quantified using the MultiSkan Go (Thermo Scientific) and analyzed using the Agilent 2100 Bioanalyzer (Agilent Technologies). For each experimental condition, two independent cDNA libraries were created and sequenced on the HiSeq 1500 (Illumina) located at the Next Generation Sequencing Core, University of Texas Medical Branch at Galveston. Enriched RNA used for cDNA synthesis was not size selected and strand-specific sequencing was performed. Each library consisted of 50bp long subsequences (reads) in a FASTQ format. Each read was assessed for its quality. Any base with a PHRED score of 15 or below was excluded. The first 14 bases of the read were trimmed and the remaining 36 bases were used for analysis. All reads mapping to human genome version GRCh38/hg38 were excluded from the analysis. The remaining rickettsial transcripts were then mapped to *R. prowazekii* strain Breinl genome (NC_020993) allowing up to two base mismatches using Bowtie2 [96]. For each prediction, the average read coverage for each nucleotide was normalized to the length of the predicted sRNA. The same was computed for 50 nucleotides up- and downstream of each prediction. The Mean Expression Value (MEV) was calculated by computing the ratio between the predicted sRNA and the flanking 50 nucleotides [53, 77]. An MEV cutoff value of ≥1.5 was used throughout this work.

### Reverse transcriptase PCR

One microgram (1μg) of DNase I treated total RNA was reverse transcribed using SuperScript^®^ VILO cDNA Synthesis Kit (Life Technologies) with random hexamers following manufacturer’s instructions. Quantitative RT-PCR was performed on StepOnePlus™ Real-Time PCR System (Applied Biosystems) using primers designed by Primer Express 3.0.1 (Applied Biosystems). For the TaqMan^®^ Assay, each 20 μL reaction contained 1X TaqMan^®^ Universal PCR Master Mix (Life Technologies), 250 nM forward primer, 250 nM reverse primer, 250 nM TaqMan probe, and 1.1 ng/μL of cDNA. Cycler conditions were: stage 1 at 50 °C for 2 min, stage 2 at 95 °C for 10 min, stage 3 (40 cycles) at 95 °C for 15 s and 60 °C for 60 s. Each TaqMan^®^ technical replicate was performed in triplicate using five biological replicates. Primers are listed in Additional file 5.

Reverse transcriptase PCRs were performed using Phusion^®^ High-Fidelity PCR Kit (New England BioLabs). Each 20 μL reaction contained a final concentration of 1X Phusion HF Buffer, 0.2μM dNTPs, 0.5μM forward primer, 0.5 μM reverse primer, 100 ng cDNA template, and 0.4 units of Phusion DNA polymerase. Thermal cycler conditions were: stage 1 at 98 °C for 30 s, stage 2 (35 cycles) at 98 °C for 15 s, 60 °C for 30 s, and 72 °C for 30 s, and stage 3 at 72 °C for 10 min. Samples were separated on a 2 % agarose gel, stained with ethidium bromide, and imaged on ChemiDoc MP imaging system (Bio-Rad). Primers are listed in Additional file 5.

## Additional files

Additional file 1:
**List of predicted sRNAs via rickettsial species.** (XLSX 595 kb) Additional file 2:
**List of target genes predicted to interact with** ***R. prowazekii*** **strain Brienl sRNAs.** (XLSX 86 kb) Additional file 3:
**Expression profile of 6S RNA (Panel a), RNaseP_bact_a (Panel b), and α-tmRNA (Panel c) during** ***R. prowazekii*** **infection of HMECs.** Y-axis denotes the nucleotide coverage. X-axis shows the genomic location of sRNAs in *R. prowazekii* genome (NC_020993). The sRNAs are shown by red arrows whereas green arrows indicate the flanking up and downstream genes. The numbers in the parenthesis indicate the exact genomic location. The flanking genes and sRNAs are not drawn to scale. (TIF 955 kb) Additional file 4:
**List of** ***Rickettsia*** **species and strains used throughout this work.** The list has been categorized into the rickettsial groups with the RefSeq ID listed in the next column. (XLSX 46 kb) Additional file 5:
**List of primers used for amplification of** ***R. prowazekii*** **predicted sRNAs.** (XLSX 49 kb)

## Abbreviations

BPROM: Bacterial Promoter Prediction Program
HGT: Horizontal gene transfer
HMECs: Human microvascular endothelial cells
IGRs: Intergenic regions
MEV: Mean expression value
ORF: Open reading frame
RMSF: Rocky Mountain spotted fever
RT-PCR: Reverse transcriptase-polymerase chain reaction
sRNA: Small ribonucleic acid
SIPHT: sRNA Identification Protocol using High-throughput Technology.

## References

[1] Wassarman KM (2007). 6S RNA: a small RNA regulator of transcription. Current Opinion in Microbiology.

[2] Papenfort K, Vanderpool CK (2015). Target activation by regulatory RNAs in bacteria. FEMS Microbiol Rev.

[3] Sharma CM, Hoffmann S, Darfeuille F, Reignier J, Findeiß S, Sittka A (2010). The primary transcriptome of the major human pathogen *Helicobacter pylori*. Nature.

[4] Gottesman S, Storz G (2010). Bacterial Small RNA Regulators: Versatile Roles and Rapidly Evolving Variations. Cold Spring Harbor Perspectives in Biology.

[5] Xiao B, Li W, Guo G, Li B, Liu Z, Jia K (2009). Identification of Small Noncoding RNAs in *Helicobacter pylori* by a Bioinformatics-Based Approach. Current Microbiology.

[6] Stazic D, Voss B. The Complexity of Bacterial Transcriptomes. J Biotechnol. 2015. http://dx.doi.org/10.1016/j.jbiotec.2015.09.041 10.1016/j.jbiotec.2015.09.041 26450562

[7] Waters LS, Storz G (2009). Regulatory RNAs in Bacteria. Cell.

[8] Wagner EGH, Romby P. Small RNAs in Bacteria and Archaea: Who They Are, What They Do, and How They Do It. In: Theodore Friedmann JCD, Stephen FG, editors. Advances in Genetics. Volume 90: Academic Press; 2015. p. 133-208. 10.1016/bs.adgen.2015.05.001 26296935

[9] Liu JM, Camilli A (2010). A broadening world of bacterial small RNAs. Current Opinion in Microbiology.

[10] Nielsen JS, Larsen MH, Lillebaek EM, Bergholz TM, Christiansen MH, Boor KJ (2011). A small RNA controls expression of the chitinase ChiA in *Listeria monocytogenes*. PLoS One.

[11] Gillespie JJ, Beier MS, Rahman MS, Ammerman NC, Shallom JM, Purkayastha A (2007). Plasmids and Rickettsial Evolution: Insight from *Rickettsia felis*. PLoS ONE.

[12] Bechah Y, Capo C, Mege J-L, Raoult D (2008). Epidemic typhus. The Lancet Infectious Diseases.

[13] Walker DH, Ismail N (2008). Emerging and re-emerging rickettsioses: endothelial cell infection and early disease events. Nature Reviews Microbiology.

[14] Dantas-Torres F (2007). Rocky Mountain spotted fever. The Lancet Infectious Diseases.

[15] Dahlgren FS, Holman RC, Paddock CD, Callinan LS, McQuiston JH (2012). Fatal Rocky Mountain Spotted Fever in the United States, 1999-2007. American Journal of Tropical Medicine and Hygiene.

[16] Raoult D, Woodward T, Dumler JS (2004). The history of epidemic typhus. Infectious Disease Clinics of North America.

[17] Mahajan SK (2012). Rickettsial diseases. J Assoc Physicians India..

[18] Parola P, Raoult D (2006). Tropical rickettsioses. Clinics in Dermatology.

[19] Civen R, Ngo V (2008). Murine Typhus: An Unrecognized Suburban Vectorborne Disease. Clinical Infectious Diseases.

[20] Andersson SG, Zomorodipour A, Andersson JO, Sicheritz-Ponten T, Alsmark UC, Podowski RM (1998). The genome sequence of *Rickettsia prowazekii* and the origin of mitochondria. Nature.

[21] Holste D, Weiss O, Grosse I, Herzel H (2000). Are noncoding sequences of *Rickettsia prowazekii* remnants of “neutralized” genes?. J Mol Evol.

[22] Gillespie JJ, Joardar V, Williams KP, Driscoll T, Hostetler JB, Nordberg E (2012). A Rickettsia genome overrun by mobile genetic elements provides insight into the acquisition of genes characteristic of an obligate intracellular lifestyle. J Bacteriol.

[23] Ogata H (2001). Mechanisms of Evolution in *Rickettsia conorii* and *R. prowazekii*. Science.

[24] McLeod MP, Qin X, Karpathy SE, Gioia J, Highlander SK, Fox GE (2004). Complete Genome Sequence of *Rickettsia typhi* and Comparison with Sequences of Other Rickettsiae. Journal of Bacteriology.

[25] Livny J (2006). Identification of 17 *Pseudomonas aeruginosa* sRNAs and prediction of sRNA-encoding genes in 10 diverse pathogens using the bioinformatic tool sRNAPredict2. Nucleic Acids Research.

[26] Livny J, Teonadi H, Livny M, Waldor MK (2008). High-Throughput, Kingdom-Wide Prediction and Annotation of Bacterial Non-Coding RNAs. PLoS ONE.

[27] Lu X, Goodrich-Blair H, Tjaden B (2011). Assessing computational tools for the discovery of small RNA genes in bacteria. RNA.

[28] Sharma R, Arya S, Patil SD, Sharma A, Jain PK, Navani NK (2014). Identification of Novel Regulatory Small RNAs in *Acinetobacter baumannii*. PLoS ONE.

[29] Altschul SF, Gish W, Miller W, Myers EW, Lipman DJ (1990). Basic local alignment search tool. Journal of Molecular Biology.

[30] Ellison DW, Clark TR, Sturdevant DE, Virtaneva K, Porcella SF, Hackstadt T (2008). Genomic comparison of virulent Rickettsia rickettsii Sheila Smith and avirulent Rickettsia rickettsii Iowa. Infect Immun.

[31] Clark TR, Noriea NF, Bublitz DC, Ellison DW, Martens C, Lutter EI (2015). Comparative genome sequencing of *Rickettsia rickettsii* strains that differ in virulence. Infect Immun.

[32] Huerta AM, Collado-Vides J (2003). Sigma70 Promoters in *Escherichia coli*: Specific Transcription in Dense Regions of Overlapping Promoter-like Signals. Journal of Molecular Biology.

[33] Mitchell JE (2003). Identification and analysis of ‘extended -10’ promoters in *Escherichia coli*. Nucleic Acids Research.

[34] Harley CB, Reynolds RP (1987). Analysis of *E.coli* Pormoter sequences. Nucl Acids Res.

[35] Walker DH (2003). Principles of the Malicious Use of Infectious Agents To Create Terror. Annals of the New York Academy of Sciences.

[36] Tjaden B (2008). TargetRNA: a tool for predicting targets of small RNA action in bacteria. Nucleic Acids Research..

[37] Wright PR, Richter AS, Papenfort K, Mann M, Vogel J, Hess WR (2013). Comparative genomics boosts target prediction for bacterial small RNAs. Proceedings of the National Academy of Sciences.

[38] Wright PR, Georg J, Mann M, Sorescu DA, Richter AS, Lott S (2014). CopraRNA and IntaRNA: predicting small RNA targets, networks and interaction domains. Nucleic Acids Res.

[39] Westermann AJ, Gorski SA, Vogel J (2012). Dual RNA-seq of pathogen and host. Nat Rev Microbiol.

[40] Wassarman KM, Storz G (2000). 6S RNA Regulates *E. coli* RNA Polymerase Activity. Cell.

[41] Shinhara A, Matsui M, Hiraoka K, Nomura W, Hirano R, Nakahigashi K (2011). Deep sequencing reveals as-yet-undiscovered small RNAs in *Escherichia coli*. BMC Genomics.

[42] Kroger C, Dillon SC, Cameron ADS, Papenfort K, Sivasankaran SK, Hokamp K (2012). The transcriptional landscape and small RNAs of *Salmonella enterica* serovar Typhimurium. Proceedings of the National Academy of Sciences.

[43] Bobrovskyy M, Vanderpool CK, Richards GR. Small RNAs Regulate Primary and Secondary Metabolism in Gram-negative Bacteria. Microbiol Spectr. 2015;3(3):MBP-0009-2014. doi:10.1128/microbiolspec.MBP-0009-2014 10.1128/microbiolspec.MBP-0009-2014 26185078

[44] Mraheil MA, Billion A, Mohamed W, Mukherjee K, Kuenne C, Pischimarov J (2011). The intracellular sRNA transcriptome of *Listeria monocytogenes* during growth in macrophages. Nucleic Acids Research.

[45] Lee E-J, Groisman EA (2010). An antisense RNA that governs the expression kinetics of a multifunctional virulence gene. Molecular Microbiology.

[46] Lenz DH, Mok KC, Lilley BN, Kulkarni RV, Wingreen NS, Bassler BL (2004). The Small RNA Chaperone Hfq and Multiple Small RNAs Control Quorum Sensing in *Vibrio harveyi* and *Vibrio cholerae*. Cell.

[47] Guillier M, Gottesman S (2006). Remodelling of the *Escherichia coli* outer membrane by two small regulatory RNAs. Molecular Microbiology.

[48] Wagner EG, Simons RW (1994). Antisense RNA control in bacteria, phages, and plasmids. Annu Rev Microbiol..

[49] Wassarman KM (2002). Small RNAs in Bacteria. Cell.

[50] Toledo-Arana A, Dussurget O, Nikitas G, Sesto N, Guet-Revillet H, Balestrino D (2009). The Listeria transcriptional landscape from saprophytism to virulence. Nature.

[51] Grieshaber NA, Grieshaber SS, Fischer ER, Hackstadt T (2005). A small RNA inhibits translation of the histone-like protein Hc1 in *Chlamydia trachomatis*. Molecular Microbiology.

[52] Albrecht M, Sharma CM, Reinhardt R, Vogel J, Rudel T (2009). Deep sequencing-based discovery of the *Chlamydia trachomatis* transcriptome. Nucleic Acids Research.

[53] Warrier I, Hicks LD, Battisti JM, Raghavan R, Minnick MF (2014). Identification of Novel Small RNAs and Characterization of the 6S RNA of *Coxiella burnetii*. PLoS ONE.

[54] Leroy Q, Lebrigand K, Armougom F, Barbry P, Thiery R, Raoult D (2010). *Coxiella burnetii* transcriptional analysis reveals serendipity clusters of regulation in intracellular bacteria. PLoS One.

[55] Hansen AK, Degnan PH (2014). Widespread expression of conserved small RNAs in small symbiont genomes. The ISME Journal.

[56] Rivas E, Eddy SR (2001). Noncoding RNA gene detection using comparative sequence analysis. BMC Bioinformatics..

[57] Washietl S, Hofacker IL, Stadler PF (2005). From The Cover: Fast and reliable prediction of noncoding RNAs. Proceedings of the National Academy of Sciences.

[58] Sridhar J, Narmada SR, Sabarinathan R, Ou H-Y, Deng Z, Sekar K (2010). sRNAscanner: A Computational Tool for Intergenic Small RNA Detection in Bacterial Genomes. PLoS ONE.

[59] Gautheret D, Lambert A (2001). Direct RNA motif definition and identification from multiple sequence alignments using secondary structure profiles. Journal of Molecular Biology.

[60] Altschul SF, Madden TL, Schaffer AA, Zhang J, Zhang Z, Miller W (1997). Gapped BLAST and PSI-BLAST: a new generation of protein database search programs. Nucleic Acids Res.

[61] Liu X, Brutlag DL, Liu JS. BioProspector: discovering conserved DNA motifs in upstream regulatory regions of co-expressed genes. Pac Symp Biocomput. 2001;6:127-38. 11262934

[62] Pain A, Ott A, Amine H, Rochat T, Bouloc P, Gautheret D (2015). An assessment of bacterial small RNA target prediction programs. RNA Biol.

[63] Macke TJ, Ecker DJ, Gutell RR, Gautheret D, Case DA, Sampath R (2001). RNAMotif, an RNA secondary structure definition and search algorithm. Nucleic Acids Res.

[64] Kingsford CL, Ayanbule K, Salzberg SL (2007). Rapid, accurate, computational discovery of Rho-independent transcription terminators illuminates their relationship to DNA uptake. Genome Biol.

[65] Argaman L, Hershberg R, Vogel J, Bejerano G, Wagner EG, Margalit H (2001). Novel small RNA-encoding genes in the intergenic regions of *Escherichia coli*. Curr Biol.

[66] Solovyey V, Salamov A. Automatic Annotation of Microbial Genomes and Metagenomic Sequences. In: Li RW, editor. Metagenomics and its Applications in Agriculture, Biomedicine and Environmental Studies. New York: Nova Science Publishers; 2010. p. 61-78

[67] Sahni SK, Narra HP, Sahni A, Walker DH (2013). Recent molecular insights into rickettsial pathogenesis and immunity. Future Microbiol.

[68] Gillespie JJ, Williams K, Shukla M, Snyder EE, Nordberg EK, Ceraul SM (2008). Rickettsia phylogenomics: unwinding the intricacies of obligate intracellular life. PLoS One.

[69] Ogata H, Renesto P, Audic S, Robert C, Blanc G, Fournier PE (2005). The genome sequence of *Rickettsia felis* identifies the first putative conjugative plasmid in an obligate intracellular parasite. PLoS Biol.

[70] Merhej V, Notredame C, Royer-Carenzi M, Pontarotti P, Raoult D (2011). The Rhizome of Life: The Sympatric *Rickettsia felis* Paradigm Demonstrates the Random Transfer of DNA Sequences. Molecular Biology and Evolution.

[71] Hindley J (1967). Fractionation of 32P-labelled ribonucleic acids on polyacrylamide gels and their characterization by fingerprinting. Journal of Molecular Biology.

[72] Faucher SP, Friedlander G, Livny J, Margalit H, Shuman HA (2010). *Legionella pneumophila* 6S RNA optimizes intracellular multiplication. Proceedings of the National Academy of Sciences.

[73] Trotochaud AE, Wassarman KM (2004). 6S RNA function enhances long-term cell survival. J Bacteriol.

[74] Weissenmayer BA, Prendergast JGD, Lohan AJ, Loftus BJ (2011). Sequencing Illustrates the Transcriptional Response of *Legionella pneumophila* during Infection and Identifies Seventy Novel Small Non-Coding RNAs. PLoS ONE.

[75] Eremeeva ME, Dasch GA, Silverman DJ (2003). Evaluation of a PCR Assay for Quantitation of *Rickettsia rickettsii* and Closely Related Spotted Fever Group Rickettsiae. Journal of Clinical Microbiology.

[76] Ozsolak F, Milos PM (2011). RNA sequencing: advances, challenges and opportunities. Nat Rev Genet.

[77] Raghavan R, Groisman EA, Ochman H (2011). Genome-wide detection of novel regulatory RNAs in *E. coli*. Genome Res.

[78] Moody MJ, Young Ra Fau - Jones SE, Jones Se Fau - Elliot MA, Elliot MA. Comparative analysis of non-coding RNAs in the antibiotic-producing *Streptomyces* bacteria. (1471-2164 (Electronic)). 10.1186/1471-2164-14-558 PMC3765725 23947565

[79] Woodard A, Wood DO (2011). Analysis of Convergent Gene Transcripts in the Obligate Intracellular Bacterium *Rickettsia prowazekii*. PLoS ONE.

[80] Gong H, Vu GP, Bai Y, Chan E, Wu R, Yang E (2011). A Salmonella small non-coding RNA facilitates bacterial invasion and intracellular replication by modulating the expression of virulence factors. PLoS Pathog.

[81] Gripenland J, Netterling S, Loh E, Tiensuu T, Toledo-Arana A, Johansson J (2010). RNAs: regulators of bacterial virulence. Nat Rev Microbiol.

[82] Masse E, Gottesman S (2002). A small RNA regulates the expression of genes involved in iron metabolism in *Escherichia coli*. Proceedings of the National Academy of Sciences.

[83] Padalon-Brauch G, Hershberg R, Elgrably-Weiss M, Baruch K, Rosenshine I, Margalit H (2008). Small RNAs encoded within genetic islands of *Salmonella typhimurium* show host-induced expression and role in virulence. Nucleic Acids Research.

[84] Fournier P-E, El Karkouri K, Leroy Q, Robert C, Giumelli B, Renesto P (2009). Analysis of the *Rickettsia africae* genome reveals that virulence acquisition in *Rickettsia* species may be explained by genome reduction. BMC Genomics.

[85] Blanc G, Ogata H, Robert C, Audic S, Suhre K, Vestris G (2007). Reductive Genome Evolution from the Mother of *Rickettsia*. PLoS Genetics.

[86] Ammerman NC, Gillespie JJ, Neuwald AF, Sobral BW, Azad AF (2009). A typhus group-specific protease defies reductive evolution in rickettsiae. J Bacteriol.

[87] Alexeyev MF, Winkler HH (1999). Membrane topology of the Rickettsia prowazekii ATP/ADP translocase revealed by novel dual pho-lac reporters. J Mol Biol.

[88] Winkler HH (1976). Rickettsial permeability. An ADP-ATP transport system. J Biol Chem.

[89] Driskell LO, Yu XJ, Zhang L, Liu Y, Popov VL, Walker DH (2009). Directed mutagenesis of the *Rickettsia prowazekii* pld gene encoding phospholipase D. Infect Immun.

[90] Whitworth T, Popov VL, Yu XJ, Walker DH, Bouyer DH (2005). Expression of the Rickettsia prowazekii pld or tlyC gene in *Salmonella enterica* serovar Typhimurium mediates phagosomal escape. Infect Immun.

[91] Crooks GE (2004). WebLogo: A Sequence Logo Generator. Genome Research.

[92] Rydkina E, Turpin LC, Sahni SK (2010). *Rickettsia rickettsii* Infection of Human Macrovascular and Microvascular Endothelial Cells Reveals Activation of Both Common and Cell Type-Specific Host Response Mechanisms. Infection and Immunity.

[93] Roux V, Rydkina E, Eremeeva M, Raoult D (1997). Citrate Synthase Gene Comparison, a New Tool for Phylogenetic Analysis, and Its Application for the Rickettsiae. International Journal of Systematic and Evolutionary Microbiology.

[94] Rydkina E, Silverman DJ, Sahni SK (2005). Activation of p38 stress-activated protein kinase during *Rickettsia rickettsii* infection of human endothelial cells: role in the induction of chemokine response. Cellular Microbiology.

[95] Rydkina E, Sahni A, Silverman DJ, Sahni SK (2007). Comparative analysis of host-cell signalling mechanisms activated in response to infection with *Rickettsia conorii* and *Rickettsia typhi*. Journal of Medical Microbiology.

[96] Langmead B, Salzberg SL (2012). Fast gapped-read alignment with Bowtie 2. Nat Methods.

